# HIV-1 dual infection is associated with faster CD4+T cell decline in a cohort of men with primary HIV infection

**DOI:** 10.1186/1742-4690-8-S2-P14

**Published:** 2011-10-03

**Authors:** Marion Cornelissen, Alexander O Pasternak, Marlous L Grijsen, Fokla Zorgdrager, Margreet Bakker, Petra Blom, Jan M Prins, Suzanne Jurriaans, Antoinette C van der Kuyl

**Affiliations:** 1 Laboratory of Experimental Virology, Dept. of Medical Microbiology University of Amsterdam, Center for Infection and Immunity Amsterdam (CINIMA), Meibergdreef 15, 1105 AZ Amsterdam, The Netherlands; 2 Department of Interna Medicine, Division of Infectious Diseases, Center for Infection and Immunity Amsterdam (CINIMA), University of Amsterdam, Meibergdreef 15, 1105 AZ Amsterdam, The Netherlands; 3 Laboratory of Clinical Virology, Dept, of Medical Microbiology, Center for Infection and Immunity Amsterdam (CINIMA), University of Amsterdam, Meibergdreef 15, 1105 AZ Amsterdam, The Netherlands

## Background

In vitro, animal and mathematical models suggest that HIV co-or superinfection would result in increased fitness of the pathogen, and possibly associated increased virulence. However, in patients the impact of HIV-1 dual infection on disease progression is unclear, because parameters relevant for disease progression have not been strictly analysed. The objective of the present study is to analyse the effect of HIV-1 dual infections on disease progression in a well-defined cohort of men having sex with men [MSM).

## Materials and methods

Between 2000 and 2009 37 primary HIV-1, subtype B infected men, with no immediate indication for combination antiretroviral therapy (cART), and sufficient follow-up were characterized with regard to single- or dual infection, HLA-I type, CCR5 genotype and coreceptor usage. Patients were followed to estimate the effect of these parameters on clinical disease progression, as defined by the rate of CD4+T-cell decline and start of cART.

## Results

Four patients presented with an HIV-1 coinfection, six patients acquired an HIV-1 superinfection, on average 8.5 months from their primary infection, and twenty-seven patients remained single infected. Longitudinal CD4+ T-cell slopes and time-weighed changes from baseline were significantly steeper in dual- as compared to single-infected patients. Multivariate analysis showed that the most important parameter associated with CD4+T-cell decline over time was dual infection (p=0.001). Additionally, patients with an HIV-1 coinfection had a significantly earlier start of cART (p<0.0001).

## Conclusions

HIV-1 dual infection is the main factor associated with CD4+ T-cell decline in untreated, primary HIV-1, subtype B infected men.

